# Applying the New Teaching Methodologies in Youth Football Players: Toward a Healthier Sport

**DOI:** 10.3389/fphys.2019.00121

**Published:** 2019-02-13

**Authors:** Antonio García-Angulo, Francisco Javier García-Angulo, Gema Torres-Luque, Enrique Ortega-Toro

**Affiliations:** ^1^Department of Physical Activity and Sport, Faculty of Sports Sciences, University of Murcia, Regional Campus of International Excellence “Campus Mare Nostrum”, Murcia, Spain; ^2^Murcia Football Federation, Murcia, Spain; ^3^Faculty of Humanities and Science Education, University of Jaén, Jaén, Spain

**Keywords:** Nonlinear Pedagogy, children, physical activity, physiology, soccer, sports initiation, rule modification

## Abstract

At early ages (6–12 years), the levels of physical activity developed in sports initiation and Physical Education often fall short of optimal levels. Ecological models of education seek, among other things, to make up for this deficit by modifying the structural elements of sport, bringing play closer to the child’s developmental characteristics. In this sense, Nonlinear Pedagogy is a model of active pedagogy that seeks the integral development of young players through a sport more in line with their abilities, and that for this is based on a system of constraints on the environment, the task and the player himself. However, there are no studies that analyze the effects of these methodologies on the parameters of physical activity at such an early age. The aim of this study was to analyze the impact of a learning methodology based on Nonlinear Pedagogy on health-related levels of physical activity (heart rate) in young football players (U-11). A quasi-experimental study was developed in which three tasks were applied using structural modifications of the football elements related to Nonlinear Pedagogy (modification of the number of players related to situations of inferiority, equality and numerical superiority; dimensions of the field of play). The sample studied was composed of football players, U-11 *n* = 32), age: 10.35 ± 0.54 years; years of experience: 2.14 ± 0.768 years. The players carried out each task for 10 min. Physical activity levels were measured by controlling heart rate using heart rate monitors (Polar Team2). The results showed very high levels of vigorous and very vigorous physical activity in all the tasks designed. These data show that the use of these new teaching methodologies has an impact on levels of physical activity in accordance with the recommended parameters.

## Introduction

Educational needs have changed over time, and so have the educational methods used (Van Wert et al., 2018). In the discipline of sport, traditional teaching styles that do not satisfy the demands and needs of sport have been replaced by Pedagogicals Models that attend to the integral development of youth athletes (Araújo et al., 2006; Jones et al., 2017; Casey and MacPhail, 2018).

Within these ecological models, Nonlinear Pedagogy is a step forward in teaching for the integral development of children (Tan et al., 2012; Chow, 2013). Nonlinear Pedagogy is a methodology of active pedagogy that is characterized by establishing a system of constraints on three aspects in the tasks: on the environment, in which all climatic, environmental, and spatial factors are taken into account (Chow et al., 2007, 2009); on the task, in terms of the modification of the number of players, playing area, playing surface or goals (Davids et al., 2007); these constraints on the task are closely related to those proposed by the Teaching Games for Understanding model (Thorpe and Bunker, 1989; Werner et al., 1996); or on the player, degree of physical development or evolutive moment (Hopper, 2002). These constraints interact with the three principles of Nonlinear Pedagogy to optimize the teaching process: (a) variability, generating tasks with a multitude of actions and movements (Davids et al., 2003); (b) self-organization, developing the capacity to react to these stimuli in order to respond successfully (McGarry et al., 2002); (c) decision-making, inasmuch as choosing the most suitable alternative for each situation is the main conditioning factor of sporting success (Araújo et al., 2006). From this idea, technical skills lose importance in relation to perceptual mechanism and decision-making (Tallir et al., 2012). These approaches are based on the need to train actions in a real context. Actions in team sports are neither predictable nor random, due to the fact that the cooperation-opposition relationship is complex (Miller et al., 2016; Moy et al., 2016; Santos et al., 2017). Several studies have demonstrated the effectiveness of this model of active pedagogy for the development of decision making and technical-tactical execution in maintaining possession of the ball and making progress in the game in football (Práxedes et al., 2018; Pizarro et al., 2019).

Among the different didactic proposals most used in Non-linear Pedagogy is the use of small-sided games (SSGs), situations in which coaches reduce different structural elements of sports. In this sense, Clemente et al. (2014), have shown that SSG significantly improve the physical condition in the sport of football.

In this line of integral child development, health-oriented sport is one of the objectives of the ecological models of sports initiation. In them, the use of new technologies plays a powerful role in terms of a more adapted sport and greater control of the health of youth players (Williamson, 2015), based on objective records. The benefits of physical activity have been widely documented in terms of improving body composition and preventing overweight, as well as metabolic and cardiovascular health (Kriemler et al., 2011; Gunter et al., 2012). Moreover, these benefits extend to psychological factors such as reduced levels of depression, stress, anxiety, and improved self-esteem and confidence (Olmedilla and Ortega, 2009; Biddle and Asare, 2011; Morgan et al., 2012). These benefits have an impact at all ages, but are especially relevant in children for proper and healthy development, prevention of disease, and improvement of cognitive functions (Hills et al., 2015; Donnelly et al., 2016).

However, on many occasions, training, and competition in collective sports are far removed from the needs and preferences of children (García-Angulo et al., 2017; Ortega et al., 2017; García-Angulo and García-Angulo, 2018). Similarly, it has been found that the time of physical exercise at healthy intensity levels is far from the recommendations in extracurricular sports activities for children (Moral, 2018). While it has been shown that adapted sports programs in school children improve parameters related to healthy body composition (Manjula et al., 2018).

In football, several studies have analyzed physiological parameters in certain training stages (Eniseler et al., 2017; Randers et al., 2017; Olthof et al., 2018), and especially in high performance sports (Owen et al., 2011). However, there are no studies that analyze the influence of the modification of the structural variables of football tasks on the parameters of physical activity in the early stages of training, when the child most requires adequate control of the loads for their development. For all the above, the aim of this study is therefore to analyze the impact of a learning methodology based on Nonlinear Pedagogy on health-related levels of physical activity (measured by heart rate) in youth football players.

## Materials and Methods

### Design

A quasi-experimental study was developed with the objective of analyzing the incidence of three tasks (small-sided games) based on Nonlinear Pedagogy on the levels of physical activity in youth football players. For this purpose three SSGs were designed in which the constraints of the task on the number of players proposed by Nonlinear Pedagogy. This study respected the ethical principles established by the UNESCO Declaration on Bioethics and Human Rights. The study was approved for development by the ethics committee of the University of Murcia (Spain) (ID 1944/2018). Following the Declaration of Helsinki the players voluntarily participated in the study and the participants’ parents/guardians signed their written consent for the development of this study.

### Participants

The sample under study was made up of players in the U-11 category (*n* = 32) who belonged to four different teams participating in federated sports initiation leagues in Spain (U-12). For the SSGs in equality and numerical superiority the sample consisted in 32 players, while for the SSGs in numerical inferiority the sample consisted in 24 players. They were 10.35 ± 0.54 years old, and 2.14 ± 0.768 years of practice.

### Instruments and Materials

For the control, monitoring and recording of the heart-rate was used the Polar Team 2^®^ system (Polar Electro, Finland), designed for the control and monitoring of both group physical activity and collective sports, was used to control and monitor heart rate parameters. Base station frequency is 2400 to 2483.5 MHz, maximum 100 mW. For the recording of the heart rate signal, Polar^®^ bands (Polar Electro, Finland) were used, which sent the data to the Polar Team 2^®^ dock using Bluetooth technology. The control, monitoring and recording system used in this study has been validated (Goodie et al., 2000) and has been used in others studies as a reference system to validate other systems (Molina-Carmona et al., 2018).

### Procedure

Three SSGs were developed in which the structural elements of the sport itself were manipulated, such as the game area and the number of players. Different numerical situations of game were introduced: (I) in situations of numerical equality, (II) in situations of numerical superiority, and (III) in situations of numerical inferiority.

The game area was modified according to the number of players participating in each SSG. For this purpose, the Regulations on sports and leisure facilities (NIDE) of the Superior Sports Council of Spain (2017) of Spain were followed, which determines the dimensions of the game spaces. Taking into account that for this age, maximum dimensions of 2925 m^2^ (65 m × 45 m) and minimum dimensions of 1500 m^2^ (50 m × 30 m) for 8-a-side football were established. The most common playing field size within these measures was taken as a reference, which is 2400 m^2^ (60 m × 40 m), which represents a playing space of 156.2 m^2^ per player. According to this game space per player and the number of players playing in each SSGs the game space in each of them was calculated. Thus the dimensions of the first SSG was 1562.5 m^2^, and the dimensions in the second and third SSG were 1406.25 m^2^.

Each of the SSGs was developed by the players in a standardized session for all groups. Before the session started, a standardized warm-up of 20 min was performed by all players. The warm-up consisted of: continuous run (3′); individual technique exercises (7′); dynamic stretching (3′); and technical and tactical situations in the official field of superiority, equality and offensive inferiority (7′).

The order of the SSGs was the same for all the teams, we started with the 5 vs. 5 numerical equality, then we made a 7′ break to rehydrate and recover the basal heart levels of the players, then we developed the second SSG of 5 vs. 4, we made a second 7′ break to rehydrate and recover the basal levels. Finally, the third SSG was developed in a 4 vs. 5 situation.

Each of the SSGs had a duration of 10′. All the SSGs were played by the same players except when the teams played in inferiority, that a player was eliminated, and that it was always the same. At the beginning of each SSG a draw was made to establish the field and the team that made the kick-off.

The standardized sessions in which the different SSGs were played at the same time for all teams and in similar weather conditions.

**FIGURE 1:**
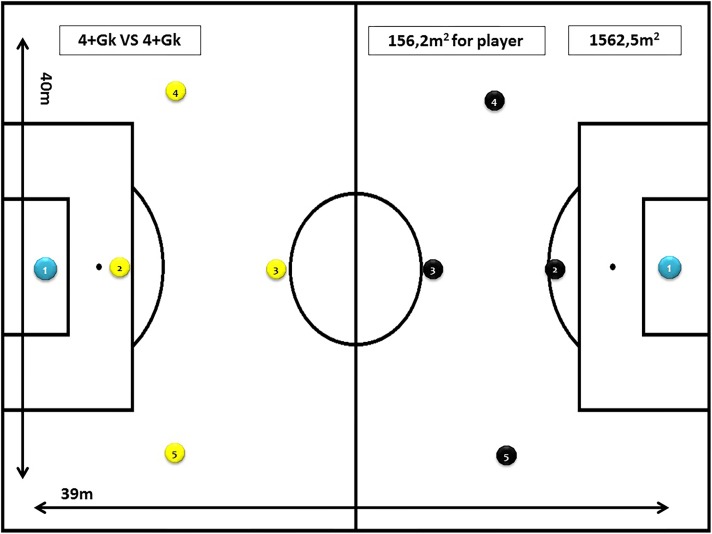
Task 1 (SSG 1). Modified task design: 4+goalkeeper vs. 4+goalkeeper (5vs5).

The levels of physical activity in each of them were recorded and analyzed using the players’ heart rate. The tasks (small-sided games) designed were as follows:

Task 1: 4+goalkeeper vs. 4+goalkeeper (5 vs. 5), for 10 min, with a space per player of 160 m^2^ (40 m × 40 m) and using the goals and balls of the category. Objective of the task: to provide the player with situations similar to the real competition (see Figure 1).

**FIGURE 2:**
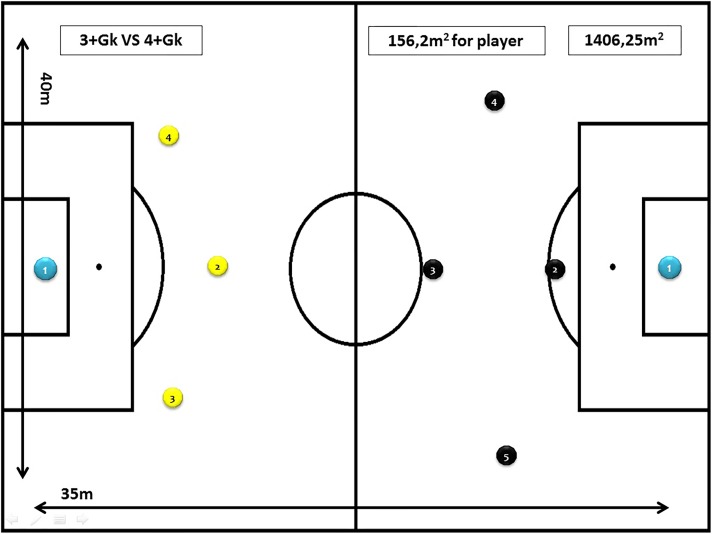
Task 2 (SSG 2). Modified task design: 3+goalkeeper vs. 4+goalkeeper (4vs5).

Task 2: 3+goalkeeper vs. 4+goalkeeper (4 vs. 5), for 10 min, with a space per player of 155 m^2^ (35 m × 40 m) and using the goals and balls of the category. Objective of the task: to facilitate defensive behavior and counter-attack situations in the game (see Figure 2).

**FIGURE 3:**
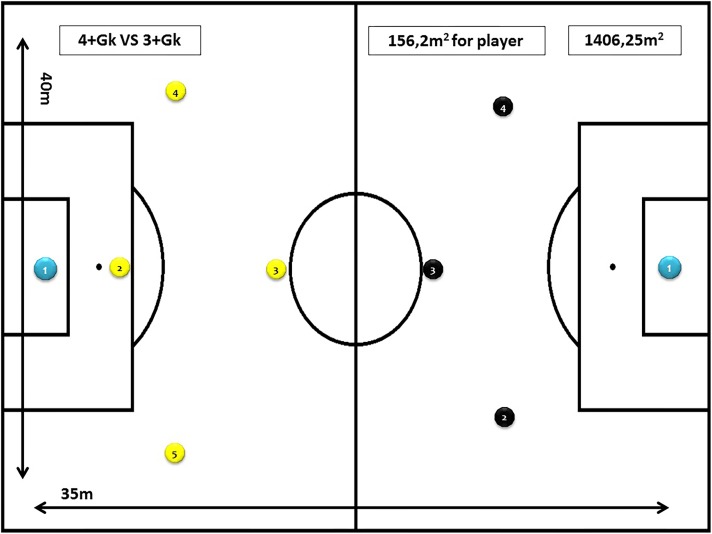
Task 3 (SSG 3). Modified task design: 4+goalkeeper vs. 3+goalkeeper (5vs4).

Task 3: 4+goalkeeper vs. 3+goalkeeper (5 vs. 4), for 10 min, with a space per player of 155 m^2^ (35 m × 40 m) and using the goals and balls of the category. Objective of the task: to facilitate and encourage the emergence of offensive actions (see Figure 3).

During the modified matches the young players wore heart rate monitors (Polar Team2). Five zones were established in accordance with the American College of Sports Medicine [ACSM] (2014), very low intensity (light): % Heart Rate Reserve (%RFC) ≤ 30, % maximum heart rate (%HRmax) ≤ 57, %VO_2max_ ≤ 37-4; low intensity (light): RFC 30-40; %HRmax 58-64; %VO_2max_ 37-45; moderate intensity: %RFC 40-59; %HRmax 65-76; %VO_2max_ 46-63; vigorous: %RFC 60-89; %HRmax 77-95; %VO_2max_ 64-90; very vigorous: %RFC ≥ 90; %HRmax ≥ 96; %VO_2max_ ≥ 91.

For each of the three tasks performed, the following variables were analyzed for each of the five heart rate zones established by the American College of Sports Medicine [ACSM] (2014): (1) Time in seconds (total time that the players remained during the tasks with a heart rate within the determined work zone); (2) Percentage of total work time (percentage of work within each of the zones established by the ACSM as a percentage of total work time).

The heart-rate of the 32 players was recorded in the situation of numerical equality (5 vs. 5) and in the situations of numerical superiority (in the situations of 5 vs. 4 and 4 vs. 5 when those players were in the team that had 5 players). Of those 32 players, 24 of them were also analyzed in the situation of inferiority (in the situations of 5 vs. 4 and 4 vs. 5 when their teams were formed by 4 players).

### Statistical Analysis

To assess possible differences between the three task analyzed, it was used an analysis of variance with repeated measures. First, the homoscedasticity of the data was checked. It was appreciated that the data met this criterion. The sphericity of Mauchley test was used, from which, it was decided to use the Trace of Pillai, or assumed sphericity (the Huynh-Feldt correction test was used, in case the principle of sphericity was not met). For *post hoc* analysis, the *post hoc* of Bonferroni was used, with a significance level of *p* < 0.05. Finally, the effect size is calculated (η^2^ or D of Cohen). For the statistical analysis the SPSS software was performed in his 24.0 version.

## Results

In Table 1, the mean values and standard deviation of the variables under study (working time, percentage of work) are recorded for each of the heart rate bands in the Task 1 (5 vs. 5), that is to say, in a situation of numerical equality.

**Table 1:** Mean and standard deviation of working frequencies in the Task 1 (5 vs. 5).

Intensity zone	Variable	Mean	Standard deviation
Zone 1 (0–57% HR_max_)	Time in seconds	12,22	29,27
	Percentage of total work time	2,02	4,87
Zone 2 (58–64% HR_max_)	Time in seconds	18,53	38,35
	Percentage of total work time	3,06	6,37
Zone 3 (65–76% HR_max_)	Time in seconds	96,25	105,80
	Percentage of total work time	15,93	17,50
Zone 4 (77–95% HR_max_)	Time in seconds	442,25	137,32
	Percentage of total work time	74,17	22,49
Zone 5 (96–100% HR_max_)	Time in seconds	33,94	80,77
	Percentage of total work time	5,68	13,50


*Players in a situation of numerical equality (I) (*n* = 32).*

The data in Table 1 showed that in zone four the youth players work the most time in the Task 1 (5 vs. 5, situation of numerical equality). Between zone 4 and zone 5 the young players during the 5 vs. 5 tasks (numerical equality) are more than 80% of the total working time.

Table 2 shows the means and standard deviation of the variables under study (working time, percentage of work) in each of the heart rate and cardiac frequency bands for the Task 2 and Task 3 (4 vs. 5 and 5 vs. 4) for players who were in a situation of numerical superiority.

**Table 2:** Mean and standard deviation of working frequencies in Task 2 and Task 3 (4 vs. 5 and 5 vs. 4).

Intensity zone	Variable	Mean	Standard deviation
Zone 1 (0–57% HR_max_)	Time in seconds	3,59	8,58
	Percentage of total work time	0,57	1,34
Zone 2 (58–64% HR_max_)	Time in seconds	8,16	21,29
	Percentage of total work time	1,28	3,25
Zone 3 (65–76% HR_max_)	Time in seconds	83,78	94,86
	Percentage of total work time	13,57	15,07
Zone 4 (77–95% HR_max_)	Time in seconds	474,69	105,79
	Percentage of total work time	77,94	18,97
Zone 5 (96–100% HR_max_)	Time in seconds	44,50	102,18
	Percentage of total work time	6,64	14,41


*Players in a situation of numerical superiority (II) (*n* = 32).*

The data in Table 2 shows that zone 4 is where the youth players who were in numerical superiority work the most time in the Task 2 and Task 3 (4 vs. 5 and 5 vs. 4), followed by zone 3. Between zone 4 and zone 5, youth players spend more than 84% of their total working time.

In Table 3, the mean values and standard deviation of the variables under study (working time, percentage of work) are recorded for each of the heart rate and cardiac frequency bands in the Task 2 and Task 3 (4 × 5 and 5 vs. 4) for players who were in a situation of numerical inferiority.

**Table 3:** Mean and standard deviation of working frequencies in Task 2 and Task 3 (4 vs. 5 and 5 vs. 4).

Intensity zone	Variable	Mean	Standard deviation
Zone 1 (0–57% HR_max_)	Time in seconds	0,71	2,03
	Percentage of total work time	0,12	0,34
Zone 2 (58–64% HR_max_)	Time in seconds	2,83	6,43
	Percentage of total work time	0,47	1,07
Zone 3 (65–76% HR_max_)	Time in seconds	63,25	88,70
	Percentage of total work time	10,51	14,74
Zone 4 (77–95% HR_max_)	Time in seconds	484,17	108,01
	Percentage of total work time	80,25	17,96
Zone 5 (96–100% HR_max_)	Time in seconds	52,42	96,04
	Percentage of total work time	8,66	15,92


*Player in a situation of numerical inferiority (III) (*n* = 24).*

Table 3 shows that zone 4 is where the youth players who were in numerical inferiority work the most time in the Task 2 and Task 3 (4 vs. 5 and 5 vs. 4), followed by zone 3. Between zone 4 and zone 5, youth players spend more than 88.9% of their total working time.

The data from Tables 1–3 show that when comparing the time in seconds between the situations of equality, superiority and inferiority, no statistically significant differences were found even in zone 2 (*F*_2,22_ = 2.538, *p* = 0.102, η^2^ = 0.187), neither in zone 3 (*F*_2,22_ = 0.864, *p* = 0.435, η^2^ = 0.073), nor in zone 4 (*F*_2,22_ = 1.730, *p* = 0.201, η^2^ = 0.136), nor in zone 5 (*F*_2,22_ = 1.069, *p* = 0.360, η^2^ = 0.089). However, trends to significance were observed in zone 1 (*F*_1.10,25.45_ = 4.093, *p* = 0.050, η^2^ = 0.151), specifically, trends to significance were observed between the situation of equality and inferiority (*p* = 0.099, *d* = 0.554).

Similarly, there were no statistically significant differences in the percentage of total working time between the situations of equality, superiority and inferiority, nor in the heart rate zone 2 (*F*_2,22_ = 2.513, *p* = 0.104, η^2^ = 0.186), neither in heart rate zone 3 (*F*_2,22_ = 0.832, *p* = 0.448, η^2^ = 0.070), nor in heart rate zone 4 (*F*_2,22_ = 1,388, *p* = 0.270, η^2^ = 0.112), nor in heart rate zone 5 (*F*_2,22_ = 0.851, *p* = 0.440, η^2^ = 0.072). However, trends to significance were observed in the heart rate zone 1 (*F*_1.05,25.42_ = 4.038, *p* = 0.052, η^2^ = 0.149), specifically, trends to significance were observed between inferiority and equality (*p* = 0.099, *d* = 0.550).

## Discussion

The aim of this paper is to analyze the impact of a learning methodology based on Nonlinear Pedagogy on health-related levels of physical activity (measured by heart rate) in youth football players. For this purpose, the constraints related to equality, superiority or inferiority in the number of players in these levels of physical activity were analyzed. It’s a novel study in terms of the age of the participants. The vast majority of researches that has analyzed the degree of physical activity in various tasks in football has focused on higher ages, adult subjects and high performance levels. This paper presents data on physiological parameters related to the intensity of physical activity at very early ages, specifically in the U-11 category. Studies of this type at this age are very rare, and most of those that have been carried out have been in school Physical Education (Davids et al., 2007; Slingerland and Borghouts, 2011; Fröberg et al., 2017; Moral, 2018). In formative football, these studies have focused on decision-making and execution (Práxedes et al., 2018), or on middle ages higher than those proposed in this paper, such as U-13 (Olthof et al., 2018); U-15 (Olthof et al., 2018); U-17 (Eniseler et al., 2017); U-18 (Sánchez-Sánchez et al., 2017), and U-19 (Özcan et al., 2018). As well as recreational adult levels (Randers et al., 2017), and higher competitive levels (Owen et al., 2011). In this sense, studies of technical-tactical actions conclude that the use of SSG helps to improve learning teaching processes (González-Víllora et al., 2011; Silva et al., 2014). On the other hand, studies that analyze physiological indicators in older soccer players to those of the object of study again indicate the usefulness of SSG for improving physical condition. In this way, the great utility of the SSG has been demonstrated, but no studies have been found in the ages object of study, nor approaches that analyze the differences or similarities between the SSG in equality, superiority, and numerical inferiority from the perspective of the heart-rate. This study allows to know the influence of the modification in the tasks in the ages not studied until now and to link the formative process with other studies that have analyzed the levels of physical activity in the tasks in soccer in higher ages.

The data found in this study showed very high levels of vigorous and very vigorous activity in the Task 1 (5 vs. 5), Task 2 (4 vs. 5) and Task 3 (5 vs. 4), with low values of moderate activity in the tasks (less than 20%) and very low values of low or very low activity (below 10%). These results may be due to the reduction in the number of players and the size of the field of play, along with the situations of numerical superiority and inferiority that are proposed in the tasks. From the player’s point of view the higher levels of physical activity may be due to the greater space for action and a greater number of interventions. Similarly, higher levels of physical activity when players are in superiority over when they are on an equal playing level may be due to the player’s moral responsibility to take advantage when his coach gives him an advantage in the task. Other studies also have analyzed the incidence of training modifications in young football players (Hill-Haas et al., 2009; Katis and Kellis, 2009). These studies have found that the reduction in the number of players in various tasks affects an increase in the physiological load, technical, and tactical parameters and the perception of the effort of the youth player in training (Hill-Haas et al., 2009; Katis and Kellis, 2009). Other studies indicate that increasing the size of the playing space has repercussions on the physiological demands of the player (Casamichana and Castellano, 2010; Belozo et al., 2018). In this same line of investigation, it has been found that the generation of numerical superiorities or inferiorities by means of supports influences the internal and external burden of youth players (Sánchez-Sánchez et al., 2017). The data in this study reinforce the data found in other studies on the importance of changes in the rules and methodology of teaching for optimal sports practice at the physical activity level (Eniseler et al., 2017; Özcan et al., 2018).

The results of this paper show high levels of vigorous and very vigorous activity in all tasks. However, there are no statistically significant differences between the three SSGs based on the Nonlinear Pedagogy, although there is a slight tendency to differences in the amount and percentage of mild physical activity. This small difference is probably due to the fact that in the 5 vs. 5 task, probably due to a question of organization, and that the number of opponents and opponents is the same, the players perceive that it is not necessary to start with a high intensity and therefore the task is started with a lower intensity, although the data indicate that it is a very short time. On the other hand, in the rest of the frequencies, the working time is very high, mainly in zones 3 and 4, and very similar in the three modalities. These data show that the reduction in the number of players is the variable that causes this high participation in medium and high intensities, although it is not so decisive if the reduction gives rise to situations of equality, superiority, or inferiority. These data show that these modifications bring the training closer to the physical activity recommendations proposed by American College of Sports Medicine [ACSM] (2014). These results are relevant as many studies indicate that levels of physical activity in training sport are below the minimum recommendations (Lonsdale et al., 2013; Hollis et al., 2016). Along the same lines, studies indicate that football generates more vigorous activity than other sports in the formative stage (Leek et al., 2011). When comparing the data from studies in which a physical activity is analyzed in an extracurricular context (sports initiation), with the data from Physical Education studies, there are large differences in favor of activities in sporting contexts (?). These differences are most likely due to the fact that extracurricular sports activities are chosen by athletes and therefore have a significant degree of motivation. However, Physical Education classes are compulsory, so that in some cases the participants do not have a motivation for the activity, and therefore the intensity of the sessions is low (Hollis et al., 2016). Physical Education teachers have a major challenge in this regard. In addition to improvements in the parameters related to physical activity with respect to Physical Education, recent studies indicate that Nonlinear Pedagogy is not limited only to improving the parameters of physical activity of young players, but is a methodology that improves decision making and technical-tactical execution in the maintenance of possession of the ball and in the advancement of the game in football (Práxedes et al., 2018; Pizarro et al., 2019).

This fact, together with the modifications proposed in this work, could be a basis to guarantee a healthy practice for children. These data reveal the need for coaches to be more aware of workloads in the different tasks they propose to their youth players. Several studies indicate that the same task may involve very different levels of workload when modified or not modified (Hill-Haas et al., 2009; Casamichana and Castellano, 2010; Belozo et al., 2018). The adequate control of the physiological loads to which the child is subjected will allow an optimization of the short training time in the formative stages and of greater benefits for the health of the child (Sánchez-Sánchez et al., 2015). Therefore, it is necessary for trainers to control the influence of the modifications introduced and their repercussion on the tasks in order to keep the tasks in activity times and intensities within the parameters recommended by the American College of Sports Medicine [ACSM] (2014), both in professional teams and mainly in teams in training stages.

The results of this study indicate that the proposed modifications maintain optimal levels of activity in all tasks. The slight differences found indicate that control and mastery of these variables by coaches is a necessary requirement for a more age-appropriate practice and for maintaining adequate levels of physical activity. However, the data should be taken with caution as it is a very small sample with very specific characteristics and ages. It is possible that an increase in the sample size generated differences between the tasks of 5 vs. 5 and the rest, in the lighter intensity bands, due surely to the fact that the players start with a moderate predisposition, since they are in situations of equality. When the players are in situations of inferiority or superiority, they perceive that it is necessary from the beginning to generate a greater intensity of work to reach the objectives. In any case, this reflection must be analyzed with caution.

The impact of the proposed methodologies on levels of healthy physical activity at other ages should be assessed. It would also be necessary to extend the study to players in the formation of elite clubs, to compare whether the impact of the proposed methodology has the same results. Future studies should assess the impact of modifying other variables, such as the inclusion of wildcards or the size and number of goals, to those already proposed and their impact on levels of healthy physical activity. In the same way it would be very interesting to evaluate the impact of the rest of the constraints on the environment and the player who proposes the Non-linear Pedagogy on the parameters of physical activity, are the heart-rate parameters different when the child plays at different times of the day? Are the levels of physical activity different when the child plays in an unusual environment? These questions may be useful for future research in this area. These data can serve as a basis for prescribing a sport closer to the levels of healthy physical activity and optimizing sports practice time.

## Conclusion

The most relevant conclusions of this study indicate that the use of the Nonlinear Pedagogy model’s own modifications based on the modification of the game space and the number of players in the sports initiation, can approximate the physical activity parameters to the levels recommended by the American College of Sports Medicine [ACSM] (2014). The effectiveness of the proposed modifications in this category (U-11) may allow coaches to design training tasks according to the physical activity needs of their youth players. These data can help overcome the low levels of very vigorous and vigorous physical activity that other studies have found in formative sport and school Physical Education.

## Author Contributions

All authors participated in the design, documentation, development, and writing of the manuscript. This paper was reviewed by all authors and all of them are responsible for its contents and providing they are responsible for the final version.

## Conflict of Interest Statement

The authors declare that the research was conducted in the absence of any commercial or financial relationships that could be construed as a potential conflict of interest.
